# Flavonoids Target Human Herpesviruses That Infect the Nervous System: Mechanisms of Action and Therapeutic Insights

**DOI:** 10.3390/v14030592

**Published:** 2022-03-13

**Authors:** Miroslava Šudomová, Kateřina Berchová-Bímová, Alena Mazurakova, Dunja Šamec, Peter Kubatka, Sherif T. S. Hassan

**Affiliations:** 1 Museum of Literature in Moravia, Klášter 1, 664 61 Rajhrad, Czech Republic; sudomova@post.cz; 2 Department of Applied Ecology, Faculty of Environmental Sciences, Czech University of Life Sciences Prague, Kamýcká 129, 16500 Prague, Czech Republic; berchova@fzp.czu.cz; 3 Department of Obstetrics and Gynecology, Jessenius Faculty of Medicine, Comenius University in Bratislava, 03601 Martin, Slovakia; alenka.liskova@gmail.com; 4 Department of Food Technology, University Center Koprivnica, University North, Trga Dr. Žarka Dolinara 1, 48 000 Koprivnica, Croatia; dsamec@unin.hr; 5 Department of Medical Biology, Jessenius Faculty of Medicine, Comenius University in Bratislava, 03601 Martin, Slovakia; kubatkap@gmail.com

**Keywords:** flavonoids, herpes simplex virus, HSV-1, HSV-2, varicella-zoster virus, Epstein–Barr virus, human cytomegalovirus, Kaposi sarcoma-associated herpesvirus, mechanisms of action, nervous system, neurological diseases

## Abstract

Human herpesviruses (HHVs) are large DNA viruses with highly infectious characteristics. HHVs can induce lytic and latent infections in their host, and most of these viruses are neurotropic, with the capacity to generate severe and chronic neurological diseases of the peripheral nervous system (PNS) and central nervous system (CNS). Treatment of HHV infections based on strategies that include natural products-derived drugs is one of the most rapidly developing fields of modern medicine. Therefore, in this paper, we lend insights into the recent advances that have been achieved during the past five years in utilizing flavonoids as promising natural drugs for the treatment of HHVs infections of the nervous system such as alpha-herpesviruses (herpes simplex virus type 1, type 2, and varicella-zoster virus), beta-herpesviruses (human cytomegalovirus), and gamma-herpesviruses (Epstein–Barr virus and Kaposi sarcoma-associated herpesvirus). The neurological complications associated with infections induced by the reviewed herpesviruses are emphasized. Additionally, this work covers all possible mechanisms and pathways by which flavonoids induce promising therapeutic actions against the above-mentioned herpesviruses.

## 1. Introduction

Human herpesviruses (HHVs) are double-stranded DNA viruses that belong to the family of *Herpesviridae* with complex virion assembly and maturation strategies [1,2]. The herpesvirus family is composed of Alpha-, Beta-, and *Gammaherpesvirinae* subfamilies, which contain diverse types of herpesvirus identified to infect humans, causing a wide range of critical diseases, ranging from cold sores to cancer [3,4]. Human alpha-herpesviruses include herpes simplex virus type 1 and type 2 (HSV-1 and HSV-2) and varicella-zoster virus (VZV or HHV-3), while human beta-herpesviruses include human cytomegalovirus (HCMV or HHV-5), HHV-6, and HHV-7. Human gamma-herpesviruses include Epstein–Barr virus (EBV or HHV-4) and Kaposi sarcoma-associated herpesvirus (KSHV), which is also known as HHV-8 [5,6,7,8].

Herpesviruses are one of the smartest infectious viruses known to humans, as they use a smart infection strategy known as ‘’run and hide’’, which means that the virus causes a primary infection and then travels to the latency site and persists for life in the infected host cells by creating latency with the ability to establish recurrent infections. This strategy reflects the smartness of these types of viruses [9,10]. Herpesviruses have a genome size of 100 to 200 kilobases, and, because of their large size, a lot remains to be revealed about how some types of these viruses transform cells [3]. Most herpesvirus infections are asymptomatic, and the disease symptoms appear once the immune system is compromised [4,11]. Since the discovery of acyclovir and other nucleoside analogs, therapeutic strategies based on these drugs for patients infected with herpesviruses remain relatively unchanged [12]. However, these approaches showed significant therapeutic outcomes, and several undesirable effects, including drug resistance, were associated with their overuse [13]. Furthermore, these medications are direct-acting antivirals that target proteins encoded by individual herpesvirus, leading to a narrow spectrum of coverage and therefore cannot address the large clinical need [14,15]. Consequently, there is an urgent need for novel antiviral drugs with diverse mechanisms of action and minimum adverse effects to overcome these obstacles [16,17]. Before the advent of synthetic compounds, injections, and expensive medications, the human body was accustomed to natural herbs and spices to fight against various diseases [18]. Plants have thousands of studies when it comes to benefiting human health as they contain a huge source of biologically active compounds [19]. Therefore, plants are considered the cornerstone of drug discovery research. Among the most studied bioactive phytochemicals with diverse biological effects are flavonoids [20,21]. More information about flavonoids, their basic structure, and bioactivities (in the context of their antiviral and neuroprotective properties) is discussed in a later section.

In this paper, we critically discuss the recent findings that demonstrated the antiviral properties of flavonoids against human herpesvirus (HHV) infections of the nervous system with a focus on the mechanisms of actions and efficient concentrations or doses. We also highlight all reported neurological diseases associated with HHV infections. Strategies/technologies that have been developed during the past five years to enhance the anti-herpesvirus properties of flavonoids are also discussed. The literature search was performed employing multiple online databases such as Web of Science Core Collection, PubMed, Scopus, SciFinder, Google Scholar, and ScienceDirect, using keywords that describe flavonoids, HHV infections of the nervous system, and the linked neurological complications. The collected data were retrieved from studies published in the years from 2017 to 2021.

## 2. Herpesvirus Infections of the Nervous System and the Innate Immune Response

Most of HHVs are neurotropic, with the ability to infect the peripheral nervous system (PNS) and central nervous system (CNS), inducing serious neurological diseases that could be monophasic, recurrent, or chronic [22,23]. HHVs-associated neurological complications, including, but not limited to, encephalitis, meningitis, and myelitis, are clinically observed in both healthy individuals and immunocompromised patients [24,25].

Epidemiologically, HHVs can trigger infections in infants, children, and adults. The transmission of infection is commonly through physical contact with the virus-infected secretions of infected patients or, in rare cases, by blood transfusion and tissue transplantation [26,27].

The infection process starts by attaching the virus to the host cell, where the host receptor recognition is a crucial step for viral infection. The attachment is mediated by numerous viral glycoproteins (located on the surface of the virion) and binding receptors. To enter the host cell, all herpesviruses were observed to require viral entry glycoproteins such as the heterodimer gH–gL and the viral fusion protein gB [28,29]. It is clinically recognized that the primary infection sites of HHVs include skin, conjunctiva, and the mucosa of the oropharynx or genitals. After the initial infection, the virus replicates and generates a hematogenous spread, termed viremia [3,30,31]. After replication, the virus exhibits latency in the latent site (at this stage, the virus is inactive) and subsequently reactivates once the host’s immune system is compromised by various physiological and environmental factors that negatively impact the immune system. On the other hand, reactivation might also occur in non-immunocompromised individuals [32,33]. Numerous experimental investigations have clinically confirmed that during latency, several viral genes are expressed, and the viral genomes frequently remain as episomes in the nuclei of infected cells and can integrate into the host genome as well [1,3,34,35]. On the other hand, all HHVs exhibit a certain amount of neurotropism, either by hematogenous spread or neuronal transmission [36].

The innate immune system takes defense actions as the first line of the defense system against the invading pathogens once the host cell detects pathogen invasion [11,37]. Innate immunity is a part of the human body defense system that uses sensors that detect viral infections. Once these sensors detect viral infection, they release an immediate alarm system that produces cytokines and interferons to impede the virus attack. Cytokines and interferons are important for activating those later immune responses such as T-cells and antibodies [38,39,40]. However, HHVs have evolved multiple mechanisms to evade the host cell’s defenses, wherein infection with these viruses substantially reshapes the gene expression landscape of the host cell by altering overall messenger RNA (mRNA) abundance or translation and repressing immune-stimulatory signals [41,42,43]. Suppression of host gene expression (termed as shutoff) can occur via various mechanisms, but one common approach is to promote degradation of mRNA [44,45]. Therefore, it is vital to figure out the strategies that HHVs might use to fight the innate immune response by reducing the levels of the innate immune sensor, proteins, and genes that could be utilized by the human body to counteract the virus. This information could help design effective therapeutic approaches against various types of HHV infections that target the nervous system [46,47].

As mentioned earlier, cytokines and interferons play a critical role in activating T-cells that are essential for triggering immune responses against various diseases, including HHV infections. Therefore, enhancing the immune responses against HHV infections by activating T-cells with natural molecules is considered a promising approach to suppress infection by HHVs. Consequently, various reports have declared that flavonoids can modulate the immune system by activating T-cells via diverse mechanisms of action and pathways [48,49,50,51].

**Figure 1:**
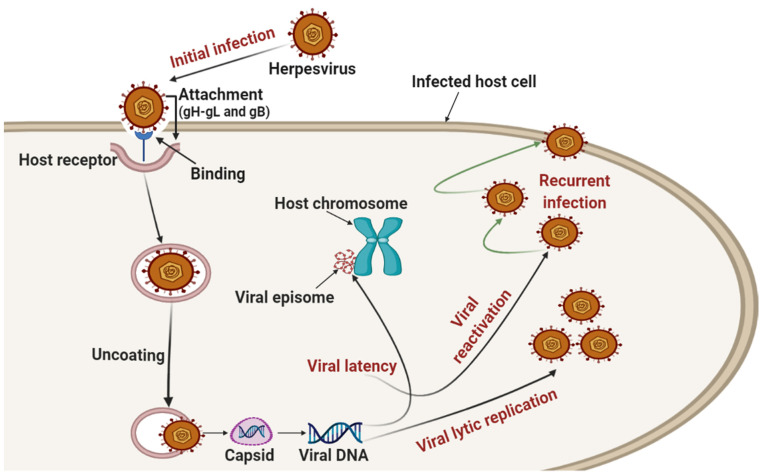
A graphical illustration displays an overview of the general steps of the human herpesvirus life cycle (initial infection, replication, latency, reactivation, and recurrent infection). Important note for readers: This illustration does not show detailed information about each step or certain types of herpesvirus. Moreover, it does not specify any component of the infected cell.

Based on the above-noted information about the general steps of the life cycle of human herpesvirus (initial infection, replication, latency, reactivation, and recurrent infection), Figure 1 depicts a general overview of the life cycle of human herpesvirus without demonstrating specific details of each step or certain types of herpesvirus.

## 3. Flavonoids: Chemical Background, Antiviral, and Neuroprotective Activities

**Figure 2:**
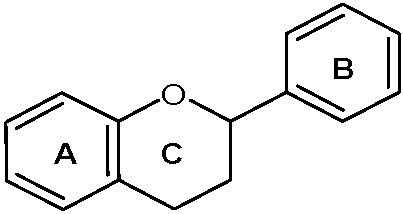
The basic structure of flavonoids.

Flavonoids are secondary metabolites that are biosynthesized by plants, including edible plants, and are commonly consumed in the human diet [52]. These metabolites are broadly distributed in the leaves, flowers, bark, and seeds of plants, and their main function is to protect plants from ultraviolet radiation, pathogens, and herbivores [53]. Chemically, flavonoids belong to the group of phenolic compounds and are mostly present in nature as glycosides that are linked to sugar in a conjugated form as monoglycosidic, diglycosidic, etc., derivatives [54,55]. Based on the chemical structure, flavonoids are categorized into six major subclasses, which are the most prevalent in the human diet, namely anthocyanidins, flavonols, flavan-3-ols, flavones, flavanones, and isoflavones [56,57]. The basic skeleton of flavonoids (Figure 2) contains 15 carbon atoms placed in two phenyl rings (A and B) joined by a heterocyclic pyran ring (C), and hydroxyl groups (one or more) are linked to the aromatic rings in free form, esterified, and/or associated with sugars [58,59]. The degree and pattern of hydroxylation, alkalization, prenylation, or glycosylation reactions impact the primary structure of the flavonoid molecule [60].

Pharmacologically, flavonoids are excellent antiviral and neuroprotective agents with proven therapeutic effects on various viral (deoxyribonucleic acid (DNA) and ribonucleic acid (RNA) viruses) and neurological diseases [61,62]. Numerous preclinical studies have revealed the ability of flavonoids to affect several steps of the viral life cycle of different viruses, including herpesviruses [63,64,65]. The potent antiviral actions of flavonoids relate to the diversity of their chemical structures. Their chemical functional groups (mainly phenyl rings, hydroxyl groups, and the associated sugar moieties) and the exact position come into play in inducing the antiviral effects [66,67,68]. Based on the inhibition mechanisms, flavonoids could be classified as inhibitors that target the viral life cycle or inhibitors that target host cell factors that affect the viral life cycle as well [15,65].

## 4. Flavonoids Target Human Herpesviruses of the Nervous System

### 4.1. Human Alpha-Herpesvirus Infections and Their Neurological Complications

According to the World Health Organization, more than 3.7 billion people worldwide suffer from HSV infections, particularly HSV-1 [69]. HSV, a prototype human alpha-herpesvirus, is categorized into two types, HSV-1 and HSV-2 [70]. HSV-1 causes oral herpes with the ability to induce genital herpes and is mainly transmitted by oral-to-oral contact, while HSV-2 is a sexually transmitted disease that primarily generates genital herpes. Moreover, HSV-2 boosts the risk of emerging and transmitting human immunodeficiency virus (HIV) infection [71,72,73]. Oral and genital infections caused by both types of HSV are mostly asymptomatic and, in some cases, can have slight symptoms that go unrecognized [74]. After primary infection, HSV replicates and induces lytic infection in epithelial cells and then penetrates the nervous system and forms a latent infection in sensory neurons [75,76]. HSV has clinically been detected to induce neurological complications in infected patients. For instance, encephalitis is the most acute neurological complication of CNS triggered by HSV-1 infection with symptoms including headache, fever, lethargy, confusion, irritability, aphasia, seizures, and focal deficit [77]. In a clinical study that has recently been published by Ordoñez et al. [78], HSV-1 was observed to contribute to the complex mechanisms of autoimmunity of Bell’s palsy, the most common acute neuropathy of cranial nerves. Infection with HSV-2 has been reported to cause various neurological complications such as encephalitis, aseptic meningitis, recurrent radiculopathy, and myelitis (particularly in immunocompromised people) [79,80].

VZV is a highly contagious human alpha-herpesvirus that causes both varicella (chickenpox) by primary infection and herpes zoster (HZ or shingles) by reactivation from latency in cranial nerve, dorsal root, and autonomic nervous system ganglia along the entire neuraxis [81,82]. VZV is latent in most ganglia, and, therefore, HZ can appear anywhere on the body; the most frequent sites are thoracic and in the cutaneous distribution of the ophthalmic branch of the trigeminal nerve. VZV is transmitted by the inhalation of aerosols from the vesicular fluid of skin lesions through direct contact with a rash and can also be spread by infected respiratory tract secretions [83,84,85]. Postherpetic neuralgia, the most common complication of HZ, is a neuropathic pain associated with HZ patients over the age of 60 years. In immunocompromised individuals, the virus can also move to the blood vessels of the brain, generating a unifocal or multifocal vasculopathy. Moreover, VZV has been observed to cause encephalitis [86,87,88]. On the other hand, in a recent retrospective monocentric clinical study, the reactivation of VZV with neurological complications was found to be triggered by coronavirus disease 2019 (COVID-19) vaccination such as the Oxford/AstraZeneca chimpanzee adenovirus-vectored COVID-19 vaccine (ChAdOx1) or the Pfizer/BioNTech BNT162b2 vaccine [89].

#### Flavonoids Target Human Alpha-Herpesviruses 

Infection with HSV and VZV is mostly acquired during childhood, when the infection rate is very high among humans. Therefore, in virology, alpha-herpesviruses are among the most studied human viruses, especially HSV [15,65]. In this section, we critically review the recent studies that have been conducted during the past five years that reveal the antiviral effects of flavonoids against alpha-herpesviruses with emphasis on the mechanisms of actions at the cellular and molecular levels and effective concentrations or doses.

Myricetin, a dietary flavone (distributed in a wide range of vegetables and fruits), was evaluated in a comprehensive study (in vitro, in vivo, and in silico) for its antiviral effects against HSV-1 and HSV-2. The results exhibited remarkable antiherpetic properties of myricetin against both viruses with multiple mechanisms at molecular and cellular levels [90]. Dihydromyricetin (DHM), a bioactive flavonoid isolated from stems and leaves of *Ampelopsis grossedentata*, has significantly blocked HSV-1 infection through multiple mechanisms evaluated by numerous virological and biochemical assays [91].

Two methoxyflavones (isolated from the aerial parts of the neotropical plant *Marcetia taxifolia)* 5,3′-dihydroxy-3,6,7,8,4′-pentamethoxyflavone (PMF) and 5-hydroxy-3,6,7,3′,4′pentamethoxyflavone (PMF-OH) were determined in vitro to have notable anti-HSV-1 activities [92]. Kim et al. [93] isolated morusin, a prenylated flavonoid, from *Mori ramulus* (the young twig of *Morus alba* L.) with anti-HSV-1 properties. Their results indicated that morusin in vitro hindered the replication of HSV-1 by reducing the expression of HSV-1 glycoprotein D (gD) protein and inhibiting HSV-1-induced reactive oxygen species (ROS). From *Morus alba* L., another research group has isolated four flavonoid compounds (kuwanon C, kuwanon T, kuwanon U, and kuwanon E) with potent antiviral properties against the replication of HSV-1 and HSV-2. The mechanism of action has been predicted by molecular docking analyses via targeting HSV-1 DNA polymerase and HSV-2 protease [17]. The anti-infectivity properties of wogonin, a bioactive flavonoid (*O*-methylated flavone), against HSV-1 and HSV-2 replications was proved by in vitro laboratory methods and its mechanisms of action were reported via modulating essential factors that are required for viral replication and altering a variety of cellular signaling pathways [94]. Rittà et al. [95] have isolated apigenin and luteolin from the chloroform extract of the leaf of *Arisaema tortuosum*, which inhibited HSV-2 replication by reducing the viral progeny production. Vitexin, an apigenin flavone glycoside, was isolated from the leaves of *Erythrina speciosa* with protective effect against HSV-1 [96].

The naturally occurring amentoflavone (broadly presents in *Biophytum sensitivum* and other plants including edible *Garcinia* species and *Juniperus communis* L.) showed substantial antiherpetic action against various strains of HSV-1, including resistant strains by blocking viral early infection via numerous mechanisms [97]. In an in vitro study, quercetin, a major constituent of the plant *Glycyrrhiza uralensis*, was investigated in vitro for its anti-infectivity properties against infected Raw 264.7 cells with HSV-1. This compound possessed potent anti-HSV-1 action via interfering with toll-like receptor-3 (TLR-3) expression (as a key mechanism) [98]. In an experiment performed to explore the effect of temperature on drug treatment against HSV-1 infection, epigallocatechin-3-gallate (EGCG), the main flavonoid molecule of green tea, demonstrated prominent inactivation of HSV-1 virions at temperatures between 25–37 °C. The study did not reveal any mechanism of action; however, the authors declared that EGCG can interfere with various steps in the HSV-1 life cycle; this is based on previously published investigations [99]. On the other hand, another research team examined the remarkable effect of EGCG on HSV-1 infection in oral epithelial cells with confirmed mechanisms of action [100]. In an animal study, the protective effect of the total flavonoids of *Ixeris sonchifolia* (Bae.) Hance (ISH) on herpes simplex keratitis (HSK) in mice was evaluated. The study outcome specified that treatment with ISH total flavonoids substantially improved the corneal lesion degree and increased mice survival rate [101]. The replication of VZV was considerably impeded by quercetin and isoquercitrin, and both flavonoid compounds were analytically detected in *Elaeocarpus sylvestris*. The suppression of VZV lytic-genes expressions were described as a mechanism of action [102]. In a combined in vitro and in vivo experiment, houttuynoid A (a bioactive flavonoid isolated from *Houttuynia cordata* Thunb.) was tested for its anti-HSV-1, anti-HSV-2, and anti-VZV activities. This compound was detected to inhibit HSV-1 infection through multiple mechanisms as well as suppress the infections of HSV-2 and VZV [103]. Houttuynoid A and houttuynoid M (isolated from *Houttuynia cordata)* were further assessed by another research team for anti-HSV-1 actions. The results showed that houttuynoid M is a houttuynoid with a bis-houttuynin chain tied to a flavonoid core, and both flavonoid compounds exhibited successful anti-HSV-1 actions [104]. Table 1 outlines a detailed description of the above-discussed antiviral effects of flavonoids along with the induced mechanisms against human alpha-herpesviruses.

### 4.2. Human Beta-Herpesvirus Infections and Their Neurological Complications

HCMV, the most studied human beta-herpesvirus, is a ubiquitous virus that causes lifelong infection in humans. It replicates in leukocytes and vascular endothelial cells and persists latently in bone marrow progenitor cells and myeloid cells [105,106]. HCMV is the most important etiological agent of congenital anomalies of the CNS induced by an intrauterine infection in humans [107]. A wide range of cell types can be infected by HCMV including neurons, radial glia, astrocytes, and endothelial cells. Furthermore, HCMV was reported to negatively impact the infected cells by disrupting neuronal proliferation, migration, and cortical cell organization [108,109].

Serious CNS damage and sensorineural hearing loss can be developed via transmitting HCMV from the placenta to the immature fetus. Other neurological complications associated with HCMV infection were reported, including seizures, microcephaly, spasticity, hypotonia, Guillain–Barré syndrome, and encephalitis [110,111,112,113].

#### Flavonoids Target Human Cytomegalovirus

The past five years have witnessed slow progress regarding the involvement of flavonoids in HCMV research. Hence, in this section, we discuss the published investigations that disclosed the anti-infectivity effects of flavonoids on HCMV with a focus on highlighting the mechanisms of actions and efficient concentration or doses.

Quercetin and isoquercitrin, identified in *Elaeocarpus sylvestris*, exhibited in vitro remarkable anti-HCMV activities by several mechanisms including suppressing the expression of HCMV immediate-early (IE) gene [102]. Deguelin, a naturally occurring flavonoid, significantly inhibited HCMV (ganciclovir-resistant strain) lytic replication by mechanisms that affect early (E) gene and protein expressions [114].

Tricin, a natural flavonoid molecule, has recently been investigated in several experiments with various mechanisms of action against HCMV. Akai et al. [115] evaluated in vitro the antiviral effect of tricin on HCMV replication. The research team also observed that infection with HCMV can promote the expression of two factors that increase HCMV infection and replication (CC-motif chemokine ligand 2 (CCL2/MCP-1) and CCR2, a CCL2-specific receptor). The results suggested that tricin notably inhibited the replication of HCMV by a key mechanism that impeded the expression of the CCL2-CCR2 axis in the HCMV replication cycle. Tricin was further investigated by another research group [116] against the replication of HCMV to reveal a new mechanism of action using various biochemical analyses. The mechanism has been elucidated through inhibiting CC-motif chemokine ligand 5 (CCL5) protein expression. It has been reported that cyclin-dependent kinase 9 (CDK9) is an essential enzyme that is required for HCMV replication. Therefore, CDK9 is a crucial target for designing new anti-HCMV drugs [117]. Accordingly, two research groups have determined in in vitro and in silico studies the anti-HCMV activities of tricin and its synthesized compound 6F-tricin (6-Fluoro-4′-hydroxy-3′,5′-dimetoxyflavone) by a mechanism via targeting CDK9 [118,119]. Table 2 presents a detailed overview of the above-reviewed antiviral effects of flavonoids along with the induced mechanisms against human cytomegalovirus.

### 4.3. Human Gamma-Herpesvirus Infections and Their Neurological Complications

Human gamma-herpesviruses consist of two tumor viruses EBV and KSHV. Both viruses share the capacity to establish primary, lytic, and latent infections in their host cells and subsequently develop various types of cancer [120,121]. However, both viruses were also described to participate in infecting the nervous system, causing various neurological diseases, especially EBV as a well-known neurotropic gamma-herpesvirus [31].

In 1964, EBV was first detected in Burkitt’s lymphoma by Sir Anthony Epstein and his research team [122,123]. This virus is best known as the cause of infectious mononucleosis (kissing disease) and can also account for CNS infections. The main transmission means is through infected saliva, and the primary infection is usually asymptomatic [124,125]. Infection with EBV causes numerous CNS complications that have been confirmed clinically such as encephalitis, meningitis, polyradiculomyelitis, cerebellitis, primary CNS lymphoma, multiple sclerosis, transverse myelitis, and cranial and peripheral neuropathies [126,127,128,129]. EBV-related CNS infections can be categorized into two groups based on the status of EBV infection, a group that includes CNS complications associated with primary or reactivated EBV infection, and the group that involves CNS complications linked with chronic active EBV infection [126,130]. Although EBV does not persist latently in neurons or other nonlymphoid cells, various experimental studies revealed that the reactivated virus can move to the CNS via infected lymphocytes, generating CNS infections at extraneural sites [130,131,132].

KSHV is the etiologic agent of Kaposi’s sarcoma (KS) and several B-cell cancers, inducing considerable morbidity and mortality in immunocompromised individuals [133]. This virus was observed to be transmitted through several means, including sex, blood, and saliva [134]. So far, limited studies have confirmed the association between KSHV and infection of the nervous system and induced neurological diseases. For instance, in a preclinical study, KSHV was proved to enter the CNS and infect neurons in HIV-positive patients [135]. Additional in vitro investigation disclosed that KSHV was effectively determined to infect human neuronal cells. The results have also revealed that KSHV might contribute to the pathogenesis of human neural diseases [136]. In a case report that introduces an HIV-1-infected patient with detected KS in the brain (caused by KSHV), neurological complications of the CNS were observed [137].

#### Flavonoids Target Human Gamma-Herpesviruses

Based on our performed literature search, we found that, over the past five years, the role of flavonoids in inhibiting essential stages in the life cycle of EBV and KSHV by diverse mechanisms of action has been documented in a few experimental investigations. Therefore, this section reviews these studies and describes all reported mechanisms of action and pathways along with effective concentrations or doses induced by flavonoids.

In an in vitro experiment performed on positive-EBV cells, two flavonoid-type compounds (luteolin-7-*O*-*β*-D-glucopyranoside and apigenin-7-*O*-[*β-*D-apiofuranosyl (1→6)-*β*-D-glucopyranoside] isolated from *Lindernia crustacea* (L.) F.Muell. (*Scrophulariaceae*)) were evaluated for their anti-EBV properties. The results disclosed that both compounds effectively hindered the EBV lytic cycle at a concentration of 20 μg/mL using immunoblot analysis. The mechanism of action of both compounds was found to be related to the inhibition of replication and transcription activator (Rta) expression [138]. Rta is an essential protein expressed by EBV during the immediate-early stage of the lytic cycle to activate the viral lytic genes [139]. In a mechanistic study, epigallocatechin-3-gallate (EGCG) was successfully observed to block EBV lytic replication (at concentrations ranging from 0.5 to 50 µM) by targeting the latent membrane protein 1 (LMP1), a key protein that plays an important role in EBV latent infection. The inhibition of EBV lytic replication by EGCG was revealed via the downregulation of LMP1 with blocking the mitogen-activated protein kinases/wild-type p53 (MAPKs/wt-p53) signal axis in AGS-EBV cells and c-Jun N-terminal kinases/c-Jun (JNKs/c-Jun) signal axis in p53 mutant B95.8 cells [140]. Treatments of EBV-positive Burkitt’s lymphoma (P3HR1) cells with protoapigenone (a protoflavone) and its analog protoapigenone 1′-*O*-isopropyl ether have efficiently inhibited the lytic replication of EBV at a concentration of 0.25 µM by impeding the expression of Rta protein with 50% inhibitory concentration (IC_50_) values of 0.127 and 0.467 µM, respectively. Moreover, protoapigenone 1′-*O*-isopropyl ether showed lower cytotoxicity (IC_50_ = 3.86 µM) than protoapigenone (IC_50_ = 34.12 µM), indicating that protoapigenone 1′-*O*-isopropyl ether is a more selective anti-EBV molecule [141]. Apigenin, a bioactive flavonoid, is widely present in various fruits and vegetables, was evaluated in a combined antiviral-and-multiple-biochemical study for its anti-EBV activity. The results declared that apigenin remarkably inhibited EBV reactivation in the lytic cycle and entirely suppressed virion production at a concentration of 50 µM. Suppressing the activities of the immediate-early gene Zta (also known as BZLF1) and Rta promoters were determined as a mechanism of action [142].

The results of a mechanistic study performed on primary effusion lymphoma (PEL) positive-KSHV cells showed that EGCG repressed the expression of Rta protein at a concentration of 50 µg/mL, demonstrating that this compound effectively suppressed the lytic replication and the viral progeny production of KSHV [143]. At various concentrations in micromolar ranges, hesperetin (a bioactive flavonoid that is broadly distributed in the genus *Citrus*) successfully inhibited KSHV lytic reactivation and decreased the production of progeny virus from the body-cavity-based lymphoma (BCBL-1) cells infected with KSHV. The inhibition of hypoxia-inducible factor 1α (HIF1α) expression was described as the mechanism of action. HIF1α is an important protein that stimulates the activity of Rta and the lytic cycle-refractory state of KSHV-infected cells [144].

**Figure 3:**
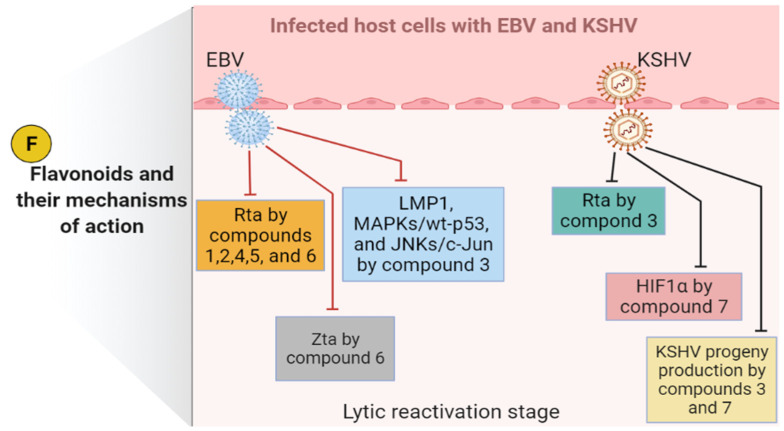
Flavonoids and their mechanisms of action against EBV and KSHV during the lytic reactivation stage of the life cycle. The blunt-end arrows indicate inhibition/downregulation. EBV, Epstein–Barr virus; F, flavonoids (luteolin-7-*O*-*β*-D-glucopyranoside (**1**), apigenin-7-*O*-[*β*-D-apiofuranosyl (1→6)-*β*-D-glucopyranoside (**2**), epigallocatechin-3-gallate (EGCG, **3**), protoapigenone (**4**), protoapigenone 1′-*O*-isopropyl ether (**5**), apigenin (**6**), and hesperetin (**7**)); HIF1α, hypoxia-inducible factor 1α; JNKs/c-Jun, c-Jun NH2-terminal kinases/c-Jun; KSHV, Kaposi sarcoma-associated herpesvirus; LMP1, latent membrane protein 1; MAPKs/wt-p53, mitogen-activated protein kinases/wild-type p53; Rta, replication and transcription activator; Zta, an immediate-early gene.

Based on the above-mentioned reviewed studies, we summarize in Figure 3 the mechanisms of action and molecular pathways induced by the reviewed flavonoid compounds against EBV and KSHV life cycles (particularly, lytic reactivation stage).

## 5. Strategies Involving Flavonoids for Enhancing Herpesvirus Treatment

During the past five years, no clinical studies were conducted to investigate the effect of flavonoids on herpesvirus diseases. Only one clinical trial involved quercetin as an ingredient of a mixed herbal product. In 2018, a clinical study was performed to test the impact of the treatment of Gene-Eden-VIR/Novirin on oral herpes, which is caused by HSV-1. Gene-Eden-VIR/Novirin is a herbal product that contains five ingredients (100 mg extract of quercetin, 150 mg extract of green tea, 50 mg extract of cinnamon, 25 mg extract of licorice, and 100 µg of selenium). The study involved 68 individuals, and each participant received treatment with Gene-Eden-VIR/Novirin (1 to 4 capsules/day) for 2 to 36 months along with standards acyclovir and valacyclovir. The study outcome revealed that treatment with the herbal Gene-Eden-VIR/Novirin notably decreased the number and duration of oral herpes outbreaks without any undesirable effects. Moreover, treatment with the herbal product was found to be more efficient and safer than treatments with acyclovir and valacyclovir [145].

Another designed study was performed on flavonoids against herpesvirus infection. Caldas Dos Santos et al. [146] have prepared a *C*-glycosylflavonoid enriched fraction of *Cecropia glaziovii* (EFF-Cg) encapsulated in PLGA nanoparticles (NP), which was designed using nonionic surfactants (poloxamer 188 (PLU). The results confirmed that the prepared formulation inhibited the replication of HSV-1 by 100% at a concentration of 80 µg/mL, with an IC_50_ value of 8.24 µg/mL, with no cytotoxic effect on Vero cells. The study concluded that EFF-Cg loaded NP displayed a promising formulation for successful drug delivery in the therapy of HHV infections.

It has been proved that combination therapy (natural products with standard antiherpesvirus drugs) can improve the treatment efficacy of HHV infections and reduce the possibility of drug resistance and cytotoxicity [4,15]. Wu et al. [100] examined the combinatory treatment of EGCG (25 µg/mL) with standard acyclovir (50 µg/mL) against infection by HSV-1 in oral epithelial cells. The combinatory treatment showed significant positive effect on HSV-1 infection, wherein the HSV-1 replication was remarkably inhibited. The intracellular viral DNA was apparently lessened in HSV-1 infected cells at 20 h post infection. Additionally, the combinatory treatment substantially suppressed the expression of viral proteins ICP5 and gD.

The molecular and physical properties of the herpesvirus are essential for regulating the infection course. The current treatment methods that use acyclovir and related nucleoside analogs target the molecular properties of the herpesvirus, particularly viral proteins [147,148]. In 2020, a research group developed in vitro a treatment approach that targets the physical properties of herpesvirus via a technique that lowers the pressure in the genome of the virus without harming the cell. Herpesvirus has internal pressure (20 atmospheres), which allows inserting its genetic materials into the cell nucleus at a high velocity after entering the host cell. Therefore, turning off the viral pressure could prevent the virus from being spread to other cells, which in turn, enables antiherpetic drugs to efficiently combat the virus [148]. Thus, flavonoids as promising anti-herpesvirus agents could participate in further studies on this developed biophysical approach.

## 6. Conclusions and Future Outlook

There are very few drugs (acyclovir and related nucleoside analogs) available to treat HHV infections; thus, when new drugs become available, especially drugs that facilitate fewer side effects with less resistance, it is a welcome discovery. Flavonoids are one of the major naturally occurring compounds that have significant medicinal actions on various human viral diseases. Therefore, in this review, we emphasized how flavonoids can help cells clear or prevent HSV-1, HSV-2, VZV, HCMV, EBV, and KSHV infections with diverse mechanisms of action at various molecular and cellular levels. Most flavonoids reviewed in the paper have been investigated in vitro, and few were examined in vivo and in silico with different mechanisms of action based on various virological and biochemical methods. Despite the protective antiviral effects generated by the reviewed flavonoids against the investigated HHVs, additional in-depth in vivo studies and human trials are highly required along with combined pharmacokinetic and pharmacodynamic investigations. Moreover, the possible side effects of flavonoids should be evaluated in additional animal and human studies.

Regarding the future directions, some recommendations might be considered in future investigations. For example, the structure–activity relationships of flavonoids are still poorly understood; therefore, further studies are greatly needed to unveil the exact functional chemical groups that are responsible for the induced anti-herpes activities. Targeting HHV infections with flavonoids might have promising therapeutic benefits, especially when combined with first-line antiherpetic drugs (as a combinatory treatment). Although numerous studies have provided insights into how HHV exploits the human cells it invades, further experiments on its Achille’s heel are needed to help develop optimal antivirals. Furthermore, understanding the full map of herpesvirus–host interactions is essential to find new approaches to combat the virus. It is necessary to design novel synthesized flavonoids that can block HHVs gene expressions by host-cell-dependent mechanisms in the early stages of infection without inducing severe side effects. More studies on nano-strategies for flavonoids delivery should be designed with promising HHV therapy.

## Figures and Tables

**Table 1:** Antiviral properties of flavonoids against alpha-herpesviruses (herpes simplex virus (type 1 and type 2) and varicella-zoster virus) with diverse mechanisms of action.

Type of Study, Method, Virus, and Cells/Animal Model	Results (Compound, Concentration, or Dose)	Mechanisms	Reference
In vitro, in vivo, and in silico. Plaque-reduction assay, CPE-inhibition assay, time-of-addition assay, various biochemical methods, and molecular-docking studies. HSV-1 and HSV-2. Vero, HeLa, and Hep-2 cells. Three-week-old female BALB/c mice.	At concentrations ranging from 2.5 to 40 µM, myricetin in vitro effectively blocked HSV-1 and HSV-2 infections by interfering with virus adsorption and membrane fusion. In vivo, treatment with myricetin at 2.5 and 5 mg/kg inhibited the infection with HSV-1 in infected mice. In silico, myricetin was found to successfully bind to HSV-2 gD protein.	Interaction with HSV-2 gD protein. Downregulation of cellular EGFR/PI3K/Akt signaling pathway.	[90]
In vitro*. * Plaque-reduction assay, virus yield reduction assay combined with several biochemical investigations. HSV-1. Vero cells.	The potent anti-HSV-1 activity of dihydromyricetin was unveiled with an EC_50_ value of 12.56 µM.	HSV-1 plaque formation and progeny virus productions were inhibited by a mechanism via the diminishment of the expression of HSV-1 IE genes (ICP4 and ICP22), early genes (ICP8 and UL42), and late genes (gB, VP1/2) at concentrations of 16 and 32 µM. Inhibition of mRNA levels of TLR9. Suppression of NF-κB and TNFα.	[91]
In vitro. Plaque-reduction assay. Vero cells.	Treatment of HSV-1-infected Vero cells with PMF and PMF-OH notably suppressed the replication of HSV-1 with EC_50_ values of 6.8 and 5.9 µM, respectively.	No mechanism of action was revealed.	[92]
In vitro. Plaque-reduction assay and multi-biochemical studies. HSV-1. Vero cells.	Morusin significantly repressed the replication of HSV-1 in infected Vero cells at 20 µM.	Inhibition of HSV-1 gD expression. Suppression of HSV-1-induced ROS.	[93]
In vitro and in silico. Plaque-reduction assay, cytopathic end-point assay, and dye-uptake method. HSV-1 (KOS strain) and HSV-2 (clinical isolates). Vero cells.	Kuwanon C, Kuwanon T, and Kuwanon U potently inhibited HSV-1 replication with IC_50_ values of 0.91, 0.64, and 1.93 µg/mL, respectively, while Kuwanon E suppressed the replication of HSV-2 with an EC_50_ value of 1.61 µg/mL.	Targeting HSV-1 DNA polymerase and HSV-2 protease by molecular docking studies.	[17]
In vitro. Cytopathic effect assay associated with various biochemical analyses. Vero and HEC-1-A cells.	At various concentrations in µM, wogonin prevented the infection of HSV-1 and HSV-2 infections by inhibiting their replication. It inhibited HSV-2-induced CPE and decreased viral mRNA transcription, viral protein synthesis, and infectious virion particle.	The mechanism of action is mediated by modulating cellular NF-κB and JNK/p38 MAPK pathways.	[94]
In vitro*. * Plaque-reduction assay, time-of-addition assay, and post-entry assay. HSV-2 and acyclovir-resistant HSV-2 strain. Vero cells.	The replication of HSV-2 was suppressed by apigenin and luteolin with EC_50_ values of 0.05 and 0.41 µg/mL, respectively, for the HSV-2 standard strain and acyclovir-resistant HSV-2 strain. EC_50_ values were found to be 2.33 and 1.55 µg/mL, respectively.	The mechanism was ascertained by decreasing viral progeny production. Apigenin was recognized to prevent cell-to-cell virus spread.	[95]
In vitro and in silico. CPE and MTT assays. HSV-1 (clinical strain). Vero cells.	Vitexin demonstrated anti-HSV-1 activity with an EC_50_ value of 18 µg/mL.	Targeting HSV-1 DNA polymerase (predicted by a molecular docking analysis).	[96]
In vitro. Plaque-reduction assay, CPE assay, and other biochemical investigations. HSV-1 (F strain; standard strain) and ACV-resistant strains (HSV-1/106, HSV-1/153, and HSV-1/Blue). Vero cells.	The considerable antiviral activities of amentoflavone were observed against HSV-1 (F strain) and ACV-resistant strains (HSV-1/106, HSV-1/153, and HSV-1/Blue) with EC_50_ values of 22.13, 11.11, 28.22, and 25.71 µM, respectively.	Suppression of viral gene production (UL54, UL52, and UL27). Inhibition of IE protein ICP0 expression. Inhibition of nuclear import of HSV-1.	[97]
In vitro. Plaque-reduction assays, western blotting, real-time PCR, and ELISA assays. HSV-1. Raw 264.7 cells and Vero cells.	The anti-infectivity action of quercetin against infected Raw 264.7 cells with HSV-1 was identified at concentrations of 10, 20, and 30 µg/mL.	Inhibition of HSV-1 gene expressions (ICP0, UL13, and UL52). Suppression of gD expression. Inhibition of TLR-3 expression. Inhibition of NF-κB and IRF3 expressions.	[98]
In vitro. Plaque-reduction assay. Wild-type HSV-1 strain (KOS1.1) Vero cells.	The virucidal activity of epigallocatechin-3-gallate against HSV-1 was revealed at concentrations as low as 1–2 µM at temperatures between 25–37 °C.	Interfering with various steps in the HSV-1 life cycle.	[99]
In vitro. Plaque assay, MTT assay, Western blotting analysis, confocal laser scanning microscopy, and real-time PCR analyses. HSV-1 (KOS strain). Oral epithelial cells and Vero cells.	Treatment of oral epithelial cells with epigallocatechin-3-gallate (25 µg/mL) significantly impeded HSV-1-induced cell death.	Reducing the expression of viral IE and ICP0 proteins. Inhibition of viral particles and viral DNA during the viral entry phase.	[100]
In vivo. CPE assay coupled with biochemical and histopathological analyses. HSV-1 (SM44 standard strain). Seventy-five adult male BALB/c mice.	Treatment with ISH total flavonoids at 50, 100, and 200 mg/kg (applied orally twice a day for two weeks) significantly boosted the corneal lesion degree and enhanced mice survival rate.	ISH total flavonoids improved the levels of IL-2 and INF-γ and lowered the levels of IL-4 in the serum of mice.	[101]
In vitro. Plaque-reduction assay with several biochemical assessments. Recombinant laboratory pOka strain of VZV (VZV–pOka). HFF cells.	Quercetin and isoquercitrin exhibited strong anti-VZV properties with IC_50_ values of 3.8 and 14.4 µg/mL, respectively.	Inhibition of VZV lytic-genes expressions. Reduction of VZV ORF62 (IE), ORF28 (E), and gB (L) transcripts levels.	[102]
In vitro and in vivo. β-galactosidase-activity assay, luciferase-activity assay, plaque-reduction assay, progeny-HSV-1-yield assay, time-of-addition assay, fusion-inhibition assay, and other biochemical methods. HSV-1 (ACV-resistant strain), HSV-2, and VZV. Vero cells and MeWo cells. Six-week-old BALB/c mice.	Houttuynoid A strongly repressed the infectivity of HSV-1, HSV-2, and VZV, with IC_50_ values of 23.50, 36.38, and 23.48 µM, respectively.	Blocking HSV-1 membrane fusion induced by viral glycoproteins (in vitro). Inhibition of HSV-1 multiplication by preventing lesion formation in a HSV-1 infection mouse model (in vivo).	[103]
In vitro. Plaque-formation assay and CellTiter-Glo Luminescent cell viability assay. HSV-1 (F strain). Vero cells.	houttuynoid M and houttuynoid A exposed anti-HSV-1 actions, with IC_50_ values of 17.72 and 12.42 µM, respectively.	No mechanism of action was defined.	[104]

ACV, acyclovir; CPE, cytopathic effect; EC_50_, 50% effective concentration; EGFR/PI3K/Akt, epidermal growth factor receptor (EGFR)-phosphoinositide-3-kinase (PI3K)-Akt; ELISA, enzyme-linked immunosorbent assay; gD, glycoprotein D; HEC-1-A cells, human endometrial cells; HSV-1, herpes simplex virus type 1; HSV-2, herpes simplex virus type 2; IC_50_, 50% inhibitory concentration; ICP, infected cell protein; IE, immediate-early; IRF3, interferon regulatory factor 3; INF-γ, interferon-γ; IL, interleukin; ISH, *Ixeris Sonchifolia* (Bae.) Hance; JNK, c-jun N-terminal kinase; MAPK, mitogen-activated protein kinase; MeWo cells, human melanoma cells; mRNA, messenger ribonucleic acid; MTT, 3-[4,5-dimethylthiazol-2-yl]-2,5 diphenyltetrazolium bromide; HFF, human foreskin fibroblasts; NF-κB, nuclear factor κB; PCR, polymerase chain reaction; PMF, 5,3’-dihydroxy-3,6,7,8,4′-pentamethoxyflavone; PMF-OH, 5-hydroxy-3,6,7,3’,4’pentamethoxyflavone; ROS, reactive oxygen species; TLR-3, toll-like receptor-3; TLR9, toll-like receptor 9; TNFα, tumor necrosis factor-α; UL27, late gene; UL52, early gene; UL54, viral immediate early gene; Vero cells, African green monkey kidney cells; VZV, varicella-zoster virus.

**Table 2:** Antiviral properties of flavonoids against human cytomegalovirus with various mechanisms of action.

Type of Study, Method, Virus, and Cells	Results (Compound, Concentration, or Dose)	Mechanisms	Reference
In vitro. Plaque-reduction assay and multiple biochemical analyses. HCMV–Towne. HFF cells.	Quercetin and isoquercitrin potently hindered the replication of HCMV with IC_50_ values of 5.9 and 1.9 µg/mL, respectively.	Inhibition of HCMV-IE gene expression. Suppression of the transcript levels of HCMV UL122 (IE), UL44 (E) and UL83 (L). Inhibition of MIEP activation by interfering with the JNK pathway.	[102]
In vitro. mCherry (a marker of infection), eGFP (a marker of late viral replication) fluorescence assays, and various biochemical analyses. Recombinant HCMV (ganciclovir-resistant strain) TB40/EmCherry-UL99eGFP. NuFF-1 cells.	Treatment of HCMV-infected NuFF-1 cells with deguelin at high (moi = 1.0) or low (moi = 0.01) multiplicities potently suppressed the HCMV lytic replication, with IC_50_ values of 55.8 and 23.4 nM, respectively.	Deguelin (250 nM) effectively repressed E and L viral gene transcriptions and reduced the expressions of IE2-86 and IE2-60 proteins.	[114]
In vitro. Plaque-reduction assay coupled with multiple biochemical tests. HCMV–Towne. HEL fibroblast cells.	Tricin suppressed the replication and infection of HCMV at a concentration of 10 µM.	Reduction of IE1 and UL54 (encoding DNA polymerase) genes expression. Inhibition of CCL2-CCR2 axis expressions in the HCMV replication cycle.	[115]
In vitro. Plaque assay combined with various biochemical analyses. HCMV–Towne. HEL fibroblast cells.	Treatment with tricin (10 µM) showed considerable inhibition of HCMV replication.	Inhibition of IE1 and UL54 gene expressions. Suppression of CCL5 protein expression.	[116]
In vitro and in silico. Plaque-reduction assay and various biochemical and molecular docking analyses. HCMV–Towne. HEL fibroblast cells.	Tricin and flavopiridol (synthetic flavonoid and standard inhibitor of CDK) exhibited notable anti-HCMV properties, with EC_50_ values of 2.09 µM and 15.8 nM, respectively.	In vitro (tricin and flavopiridol repressed the activity of CDK9, with IC_50_ values of 1.38 µM and 8.20 nM, respectively). The anti-CDK9 activity of tricin is related to the phosphorylation of the carboxy-terminal domain of RNA polymerase II. In silico (tricin was found to bind to the ATP-binding site of CDK9).	[118]
In vitro and in silico. Plaque-reduction assay and multiple biochemical and molecular docking simulations assays. HCMV–Towne. HEL fibroblast cells.	The anti-HCMV activities of tricin and 6F-tricin were determined, with EC_50_ values of 54.3 and 0.13 nM, respectively.	In silico (tricin and 6F-tricin were detected to bind to the ATP-binding site of CDK9, and significant binding affinity was observed with 6F-tricin).	[119]

CCL2, CC-motif chemokine ligand 2; CCL5, CC-motif chemokine ligand 5; CCR2, a CCL2-specific receptor; CDK9, cyclin-dependent kinase 9; EC_50_, 50% effective concentration; HCMV, human cytomegalovirus; HEL, human embryonic lung; HFF, human foreskin fibroblasts; IC_50_, 50% inhibitory concentration; IE, immediate–early; JNK, c-jun N-terminal kinase; MIEP, major IE (MIE) enhancer/promoter; moi, multiplicity of infection; NuFF-1, primary newborn human fibroblasts.

## Data Availability

Data are available throughout the manuscript.
